# Search of ligands suitable for ^212^Pb/^212^Bi in vivo generators

**DOI:** 10.1007/s10967-012-2238-4

**Published:** 2012-09-19

**Authors:** Barbara Bartoś, Krzysztof Lyczko, Agata Kasperek, Seweryn Krajewski, Aleksander Bilewicz

**Affiliations:** Centre for Radiochemistry and Nuclear Chemistry, Institute of Nuclear Chemistry and Technology, Dorodna 16 03-195, Warszawa, Poland

**Keywords:** In vivo generator, ^212^Pb, ^212^Bi, Polyaminipolycarboxylate ligands

## Abstract

The short half-life of ^212^Bi and ^213^Bi limits the application of these radionuclides in α radionuclide therapy. The labeling of biomolecules with ^212^Pb (mother nuclide of ^212^Bi) instead of ^212^Bi or ^213^Bi has the advantage of obtaining a conjugate with a half-life of 10.6 h, compared with of 60 min for ^212^Bi or 46 min for ^213^Bi. Previous attempts to prepare a potential in vivo generator with ^212^Pb complexed by the DOTA chelator failed, because about 36 % of Bi was reported to escape as a result of the radioactive decay $^{{212}}{\text{Pb}}{\mathop{\longrightarrow}\limits^{\beta ^{ - }}}{^{212}{\text{Bi}}}$. Herein, we report studies on the stability of the ^212^Pb complexes with eight selected polydentate ligands, which demonstrate high affinity for 3+ metal cations. From the ligand studied DOTP and BAPTA show a sufficient ^212^Pb labeling yields but only ^212^Pb–DOTP complex is stable in isotonic solution of sodium chloride making this way radioactivity level of released ^212^Bi is below the limit of detection. It should be emphasized that the DOTP complex is stable only in the case when the concentration of free DOTP exceeds 10^−4^ M.

## Introduction

In the field of targeted radiotherapy, the selection of radionuclide depends on the type of the treated disease. Solid tumors are generally treated with high and medium energy *β* ^−^-emitters such as ^90^Y, ^188^Re and ^131^I, because their *β* ^−^-particles have a tissue range of several millimeters. The effective tissue range of *β* ^−^-particles is not optimal for treatment of tumors forming small clusters of cells and for treatment of single cancer cells and micrometastases. Treatment of these neoplastic diseases could be more effective with α-emitters, which combine short range with high linear energy transfer, combination that results in the relatively high biological effect and cytotoxicity [1]. Owing to this, α-particles are able to make lethal double strand breaks in DNA. When the double stranded DNA molecules breaks, there is very little chance to repair such damage. Humm and Cobb [2] reported that to attain single cell kill probability of 99.99 % tens of thousands of β-decays at the cell membrane are required, whereas in the case of α-emitters only few α-decays at the cell membrane are sufficient to kill malignant cells. Due to high radiotoxicity of α-particles, high degree of accuracy is required to deliver the radiation to the target cells without targeting normal cells. From the medical point of view, α-particles can be used either for treatment of cancer micro-metastasis, or to destroy tumor margins after surgical resection. Another potential application is in treating cancers such as lymphoma and leukemia, which are present as free-floating tumor cells in the circulation system [3]. Till now, only few clinical studies with ^213^Bi and ^211^At labeled peptides and monoclonal antibodies have demonstrated the potential of alpha particle emitting isotopes in radionuclide therapy [4, 5].

There are only few α-particle emitting radionuclides, which have properties suitable for developing therapeutic radiopharmaceuticals: generator-obtained ^212^Bi (*t*_1/2_ = 60 min), ^213^Bi (*t*_1/2_ = 46 min), ^226^Th (*t*_1/2_ = 30 min), ^225^Ac (*t*_1/2_ = 10 days), ^227^Th (*t*_1/2_ = 18.7 days), as well as the cyclotron-produced ^211^At (*t*_1/2_ = 7.2 h). The available α-emitters have serious shortcomings, because in the case of ^225^Ac and ^227^Th the designed ligand must form chemically stable complexes with both parent and decay radionuclides. ^225^Ac decays directly to ^221^Fr (alkali metal), which has a half-life of 4.9 min and escapes from ^225^Ac-radiobioconjugate. Similar situation appears in the case of ^227^Th, where the decay product, the gaseous ^219^Rn, easily liberates itself from ^227^Th-radioconjugate. Application of ^211^At is limited, because astatine as the heaviest halogen forms weak bond with a carbon atom in the biomolecule. Therefore, ^211^At-bioconjugates are unstable under physiological conditions.

In the case of ^212^Bi, ^213^Bi and ^226^Th short half-life often limits the application of these nuclides to situations when the tumor cells are rapidly accessible to the targeting agent. However, the short half-life of ^212^Bi could be effectively lengthened by chelation of the parent ^212^Pb radionuclide (*t*_1/2_ = 10.6 h) to a biomolecule [6]. In comparison with direct use of ^212^Bi, radiopharmaceuticals based on ^212^Pb would have much broader applicability, because the half-life of ^212^Pb corresponds better with the pharmacokinetics of various biomolecules. Moreover, the ^212^Pb–^212^Bi in vivo generator delivers the dose per unit of administered activity ten times greater than that in the case of ^212^Bi alone or of the ^213^Bi α-emitter [7]. Thus, the required activity of the radiopharmaceutical preparation would be greatly reduced, and making this way generation and administration of the α-emitting radiopharmaceutical much easier.

It is very important that ^212^Bi formed in the *β* ^−^-decay of ^212^Pb remains bound to the carrier. This is because free bismuth localizes in the kidneys, prohibiting this way the use of structures that are not effective in stabilizing ^212^Bi in vivo [8]. In theory, the decay of ^212^Pb should not generate a problem with retention of ^212^Bi. The calculated recoil energy of the Bi nucleus is only about 0.5 eV. This is not sufficient to break a chemical bond, which requires about 10 eV. However, over 30 % of the γ-rays emitted when ^212^Pb decays are internally converted during the decay time. The resulting cascade of conversion electrons brings ^212^Bi to highly ionized states such as Bi^5+^ and Bi^7+^, hence the energy required to neutralize the charge is sufficient to break chemical bonds [9]. The potential use of ^212^Pb as an in vivo generator has been studied in earlier works [8, 10, 11]. Previous attempts to prepare a potential in vivo generator with ^212^Pb complexed by the DOTA chelator [11] failed, because about 36 % of Bi was reported to escape as a result of the radioactive decay ${}^{{^{ 2 1 2} }}{\text{Pb}}\mathop{\longrightarrow}\limits^{{\beta^{ - } }}{}^{{^{ 2 1 2} }}{\text{Bi}}$. Because the free highly energetic radiobismuth escapes from the complex during the decay, toxicity emerges when unchelated ^212^Bi accumulates in various organs, mainly in kidneys. Formation of kinetically inert Bi^3+^–DOTA complexes is very slow, therefore liberated ^212^Bi very poorly reassociates with DOTA.

In this paper we report the formation and stability studies of ^212^Pb complexes with various polydentate ligands exhibited faster than DOTA kinetics of complex formation.

## Experimental

### Lead-212

The 1 MBq of ^212^Pb (*t*_1/2_ = 60 min) was obtained from ^232^U as one of the decay products. Separation of ^212^Pb from ^232^U and other decay products was performed in a two-step procedure. In the first step, ^224^Ra was eluted by 0.1 M HNO_3_ from HDEHP-Teflon column loaded with ^232^U. In the second step ^212^Pb was separated from ^224^Ra on cation exchange resin Dowex 50 × 8 by elution with 1.0 M HCl. The effluent was acidified with HNO_3_, evaporated and the residue was dissolved in 0.01 M HNO_3_.

### Measurements

The radioactivity was measured by γ-spectrometer using the HPGe detector (Canberra) with associated electronics (resolution 2.09 keV for 1,332 keV ^60^Co line, efficiency ca. 30 %), coupled to the multichannel analyzer TUKAN (The Andrzej Soltan Institute for Nuclear Studies, Świerk, Poland).

### Ligands

We have chosen the following acyclic ligands for the studies: 8-dentate diethylenetriaminepentaacetic acid (DTPA), 6-dentate *N,N*-bis(2-hydroxybenzyl)ethylenediamine-*N,N*-diacetic acid (HBED), 6-dentate 1,2-bis(*o*-aminophenoxy)ethane-*N,N,N’,N’*-tetraacetic acid (BAPTA), 8-dentate ethylene glycol-bis(2-aminoethylether)-*N,N,N′,N′*-tetraacetic acid (EGTA) and 10-dentate triethylenetetraamine-*N,N′,N″,N″′*-hexaacetic acid (TTHA). From the cyclic ligands we have chosen 8-dentate 1,4,7,10-tetraazacyclododecane-1,4,7,10-tetraacetic acid (DOTA), 8-dentate 1,4,7,10-Tetraazacyclododecane-1,4,7,10-tetrayl-tetrakis(methylphosphonic acid) (DOTP) and 6-dentate 1,4,7-triazacyclononane-1,4,7-triacetic acid (NOTA).

### Synthesis of radiolabeled complexes

The experimental conditions for labeling, such as the metal-to-ligand molar ratio, pH, time of reaction and temperature, were optimized to achieve a high complexation efficiency. The ^212^Pb complexes with the studied ligands were synthesized by mixing 50 μl of non-carrier-added ^212^Pb in 0.01 M HNO_3_ with 5 μl of either 10^−1^ or 10^−2^ M solution of the respective ligand. The volume of solution was adjusted to 500 μl by adding 0.01 M CH_3_COONH_4_ solution, and pH were settled at pH 6 or 7 using 2 M NaOH. Complexes with acyclic ligands were prepared at room temperature in 2 h.

### Determination of labeling efficiency and assay

The determination of labeling efficiency was achieved in accordance with the modified procedure proposed by Mirzadeh et al. [9] by isolation of uncomplexed cations by the use of chelating Chelex 100 resin in a small column (*d* = 3 mm, *h* = 10 mm). In preliminary experiments we found that when solution containing nca ^212^Pb and ^212^Bi was loaded on the column all activity remained on the column, even after elution with 0.1 M NH_4_NO_3_. In next step the ^212^Pb and ^212^Bi radionuclides were quantitatively eluted with 2 ml of 5 M HCl. We assumed that under the same conditions the negatively charged complexes of Pb^2+^ and Bi^3+^ would be eluted from the column by 0.1 M NH_4_NO_3_. This separation procedure was tested on Pb and Bi complexes formed by 0.01 M DOTA and DTPA ligands, and we found that these complexes were completely eluted by 2 ml of 0.1 M NH_4_NO_3_.

### Assay of ^212^Bi after decay of ^212^Pb–L complexes

The complexes were prepared as described above. Concentration of the synthesized complexes was decreased using isotonic solution of sodium chloride (0.9 % NaCl solution), in order to obtain 0.5 ml samples. Solutions were incubated for 4 h to attain ^212^Pb–^212^Bi radioactive equilibrium and then in order to separate complexes from the uncomplexed cations the solution was loaded on the column filled with Chelex 100 resin (3 × 10 mm). To achieve the separation the column was washed with 2 ml of 0.1 M NH_4_NO_3_ solution which eluted the complexes. The retained uncomplexed ^212^Pb and ^212^Bi cations were next eluted with 2 ml of 5 M HCl. The activities of the eluted fractions were measured over 5 h time period.

## Results and discussion

The labeling of biomolecules with ^212^Pb instead of ^212^Bi or ^213^Bi has the advantage of obtaining a conjugate with a half-life of 10 h, instead of 60 min for ^212^Bi or 46 min for ^213^Bi.

Therefore, when ^212^Pb labeled conjugate is used, the delivered dose is much greater per unit of administered activity than in the case of ^212/213^Bi conjugates [7]. As noted in [12] a dose of 10 mCi of ^212^Pb was equally effective as a 500 mCi injected dose of ^213^Bi. However, as reported by Mirzadeh et al. [9] and Miao et al. [13] approximately one-third of the radioactivity escaped from the DOTA chelator due to ionization associated with the decay of ^212^Pb to ^212^Bi. In the case of radiobioconjugate Fu-Min Su et al. [14] found that ^212^Pb–DOTA-biotin was initially stable, but 30 % of ^212^Bi activity was released from the DOTA-biotin in 4 h. This result is in agreement with that reported by Mirzadeh et al. [9] who found that 36 % of ^212^Bi activity was released from ^212^Pb–DOTA in the decay.

Redistribution was not a concern for ^212^Pb internalized in tumor cells, since diffusion of metal ions across the cell membrane would be very slow. However, loss of ^212^Bi from circulating ^212^Pb-bioconjugate could allow ^212^Bi to redistribute and irradiate normal organs.

**Fig. 1:**
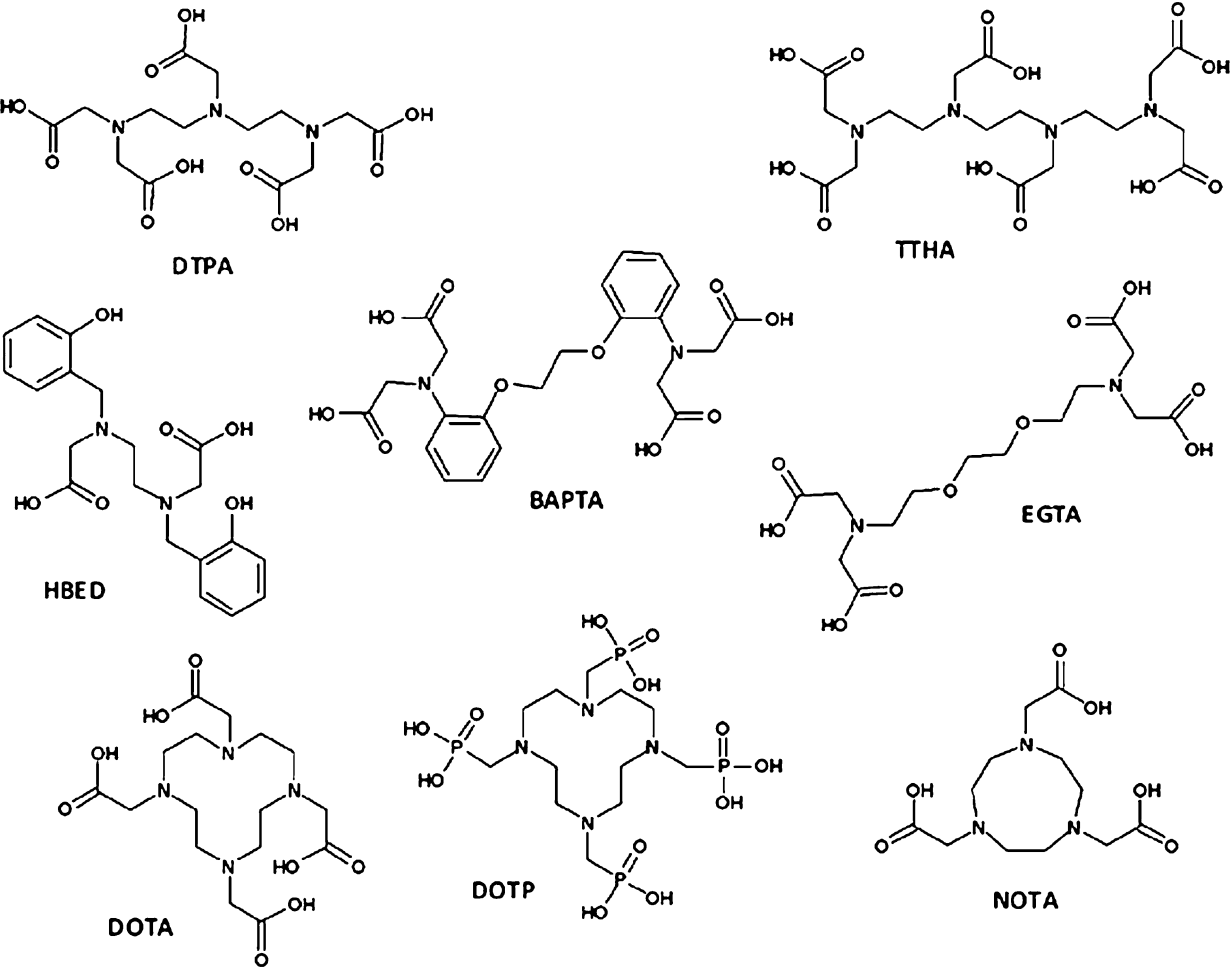
Structure of the ligand studied

In the previous studies, DOTA and its *N*,*N*,*N*,*N*-tetraamide analog were used for binding ^212^Pb to biomolecules [11]. In our opinion, because formation of kinetically inert Bi^3+^–DOTA complex is very slow, the released ^212^Bi from the ^212^Pb–DOTA complex very poorly reassociates with DOTA. In our studies, we examined selected acyclic and cyclic polyaminopolicarboxylate ligands, which form complexes with bismuth cations more rapidly than does DOTA. The ligands demonstrating high affinity for 3+ metal cations like Fe^3+^ and lanthanides were selected for our studies. The structure of the ligands is presented in the Fig. 1.

From the studied ligands DOTP and BAPTA are the only two, which can be taken into consideration for designing new applicable radioconjugates, because they demonstrate sufficient labeling yields Table 1. The high yield of labeling can be achieved only in the case, when the ligand concentration exceeds 10^−4^ M. The remaining ligands form complexes with ^212^Pb with too low efficiency. Therefore, only the ^212^Pb–DOTP and ^212^Pb–BAPTA complexes were selected for studying stability in isotonic solution of sodium chloride (0.9 % NaCl).

**Table 1:** Labeling yield of the ligands by ^212^Pb. Solution—in 0.01 M CH_3_COONH_4_, pH = 6

Ligand (concentration M)	Labeling yield (%)
DOTP (10^−4^ M)	89.5
DOTP (10^−5^ M)	62.7
DTPA (10^−4^ M)	47.2
TTHA (10^−4^ M)	68.3
EGTA (10^−4^ M)	73.1
BAPTA (10^−4^ M)	85.8
HBED (10^−4^ M)	27.2
NOTA (10^−4^ M)	<10

As shown in Table 2 the ^212^Pb–DOTP complex is stable in isotonic solution of sodium chloride, because at DOTP concentration of 10^−4^ M only very small amount of ^212^Pb escapes into solution. The radioactivity level of released ^212^Bi is under the limit of detection. Comparison of our results with those on ^212^Pb–DOTA, described by Mirzadeh et al. [9], shows that DOTA forms with ^212^Pb kinetically inert complexes. Unfortunately, ^212^Bi the decay product of ^212^Pb, released to solution very poorly reassociates with DOTA. On the contrary, DOTP forms with ^212^Pb more labile complexes, for which the escaped ^212^Bi easily reassociates with the ligand. It should be emphasized that ^212^Pb–DOTP is stable only in the case when concentration of the free ligand exceeds 10^−4^ M.

**Table 2:** Stability of ^212^Pb-DOTP and ^212^Pb-BAPTA complexes in isotonic solution of sodium chloride

Ligand	Ligand concentration (M)	Free ^212^Pb activity (%)	Free ^212^Bi activity (%)
DOTP	10^−4^	2	0
	10^−5^	20	0
BAPTA	10^−4^	80	70

The activity of the ^212^Pb-DOTP solution was 2.6 × 10^4^ cpm and that of ^212^Pb-BAPTA 2.5 × 10^4^ cpm

The results obtained show that DOTP could be used as a ligand in designing ^212^Pb/^212^Bi in vivo generators, but only in the case when high specific activity of the radiopharmaceutical is not required, as it happens in palliation therapy of bone metastasis.
